# Quantitative Evaluation of the Echo Intensity of Paraneural Area and Myofascial Structure around Median Nerve in Carpal Tunnel Syndrome

**DOI:** 10.3390/diagnostics10110914

**Published:** 2020-11-08

**Authors:** Chenglei Fan, Caterina Fede, Carmelo Pirri, Diego Guidolin, Carlo Biz, Veronica Macchi, Raffaele De Caro, Carla Stecco

**Affiliations:** 1 Department of Neurosciences, Institute of Human Anatomy, University of Padua, 35121 Padua, Italy; yutianfan1218@163.com (C.F.); caterina.fede@unipd.it (C.F.); carmelop87@hotmail.it (C.P.); diego.guidolin@unipd.it (D.G.); veronica.macchi@unipd.it (V.M.); rdecaro@unipd.it (R.D.C.); 2 Department of Surgery, Oncology and Gastroenterology, Orthopedic Clinic, University of Padua, 35128 Padua, Italy; carlo.biz@unipd.it

**Keywords:** paraneural area, myofascial structure, echo intensity, median nerve, carpal tunnel syndrome, ultrasound

## Abstract

The aim of this study was to investigate whether the echo intensity (EI) of the paraneural area (PA), the median nerve (MN) at the carpal tunnel, the EI of the myofascial structure (MS) around MN, the ‘PA and MN’ at the mid-forearm, and the MN transversal displacement at both sites differs between persons with carpal tunnel syndrome (CTS) and control subjects. Methods: In total, 16 CTS patients and 16 controls, age- and gender-matched, were recruited. Cross-sectional ultrasound images of MN were obtained to evaluate the EI of the PA, the MN at carpal tunnel, the EI of MS, and the ‘PA and MN’ at the mid-forearm in a natural position, then images were taken after a whole-hand grasp movement, to evaluate MN transversal displacement. Inter-rater and intra-rater reliability in control, and differences in the EI and MN displacement between CTS and control, were analyzed. In addition, the correlations between ultrasound parameters and MN displacement were evaluated. Results: The quantitative EI of PA, MN, EI of MS, ‘PA and MN’ had high inter-rater and intra-rater reliability in the control. The EI of PA, MS and ‘PA and MN’ were significantly higher in CTS subjects (*p* < 0.01), whilst there was no significant difference in the EI of MN at the carpal tunnel. MN displacement was significantly decreased both at the carpal tunnel and the mid-forearm in CTS subjects (*p* < 0.01). In addition, there were negative correlations among the EI of PA (rs = −0.484, *p* = 0.004), EI of MS (rs = −0.479, *p* = 0.002), EI of ‘PA and MN’ (rs = −0.605, *p* < 0.001) and MN transversal displacement. Conclusions: The higher EI of PA and MS around MN in CTS may indicate greater fibrosis along the course of MN, reducing fascial adaptability, influencing the synergy and coordination of the MS, and increasing the shear stress between MS and MN, and it may further increase the abnormal pressure on the MN not only at the carpal tunnel, but also at the mid-forearm. These results may partly explain the role of PA and MS in CTS pathogenesis.

## 1. Introduction

The pathophysiological mechanisms of carpal tunnel syndrome (CTS) involved in median nerve (MN) compression and traction are thought to be complex. Even if the carpal tunnel is, by far, the most common compression location, other locations are possible, and several compression sites can be associated with the generation of a double crush syndrome (DCS), which is a distinct compression at two or more locations along the course of a peripheral nerve, that can coexist and synergistically increase symptom intensity [1]. This phrase was originally coined by Upton and McComas in 1973 [2], “as a means of explaining how one site of injury on a nerve made that nerve more susceptible to injury at another location”. In addition, the MN is not an isolated structure but is entirely connected to myofascial structures, as demonstrated by our previous histological study [3]. In this study we demonstrated that the paraneural area is in continuity with the deep fasciae of the forearm, suggesting that an unbalanced tension of epimysial fasciae can affect the paraneural area, limiting nerve displacement, and consequently this must be included in CTS pathogenesis [3]. Therefore, the surrounding fascial structures of the peripheral nerve have been increasingly highlighted recently as possible causes of nerve entrapment [4].

Echo intensity (EI) is the mean pixel intensity of a specific region of interest from ultrasound (US) images [5]. Changes in EI are believed to be caused by intramuscular fibrous tissue and/or adipose tissue [6,7]. Studies have demonstrated that interstitial fibrous tissue causes increased muscle echo intensity (EI) [8,9]. In addition, the reliability of EI measurements and their utility for the musculoskeletal system have been confirmed by other researchers [10,11].

The aim of this study was therefore to ascertain whether the EI of the paraneural area (PA), the MN at the carpal tunnel, the EI of the myofascial structure (MS) around the MN, and the EI of ‘PA and MN’ upon mid-forearm and MN transversal displacement at both segments differ between persons with CTS and control subjects, and whether alterations in these US parameters are related to the MN transversal displacement.

## 2. Materials and Methods

### 2.1. Participants

This study was completed in accordance with the Helsinki Declaration as revised in 2013. Informed consent was obtained from all research subjects before they participated in the study. Ethics Approval for the histological study was obtained from the ethical committee of the Institute of Anatomy of the University of Padua.

US was evaluated in 16 CTS patients experiencing severe pre-surgical CTS, who were matched with 16 control subjects for age and gender between January 2018 and January 2020. We recruited only severe CTS patients, according to the grading described by Sucher BM., (2013) [12], who were confirmed by electrodiagnostic diagnosis. Patients were excluded if they had medical conditions that could cause peripheral neuropathy, such as diabetes mellitus, malignancy and other endocrine diseases. The age- and gender-matched control subjects without health problems were recruited. The control subjects were proven to be CTS negative using clinical tests (Gilliat test, Phalen test, Tinel pseudo-test) and electrodiagnostic tests.

### 2.2. Setting of the Ultrasound Machine

US examinations were measured on an Esaote MyLab Seven B-mode US machine (Esaote SpA, Genova, Italy) with 37-mm linear array transductors, at 6–18 MHz. The same equipment settings were used in all subjects so as to not affect the measurements of EI for the musculoskeletal structures, and were the following: 50 dB gain, 56 dB dynamic range, 2 cm depth at carpal tunnel and 3.5 cm depth at mid-forearm, which were considered adequate for imaging the MN and the surrounding tissue. In addition, to minimize anisotropy (angular dependence), the probe was also aligned so that it was perpendicular to the scaphoid, the pisiform tubercle and the MN. To minimize tissue compression and variability between neutral and flexed positions, the probe was kept perpendicular and firm [11,13].

### 2.3. Inter-Rater and Intra-Rater Reliability in Control Subjects

To assess test–retest reliability, the intra-class correlation coefficient (ICC A,1: two-way random, single measures, absolute agreement) was used to quantify inter-rater and intra-rater reliability for the quantitative EI of PA, the MN at the carpal tunnel, the EI of MS, and the ‘PA and MN’ at the mid-forearm in the control group. To assess the inter-rater reliability of quantitative US, two experienced medical doctors (one with 3 years’ experience in musculoskeletal US imaging, and another one with 2 years’ experience in US imaging) conducted US examinations in each control subject on the same day. To assess intra-rater reliability, six images per subject were taken by one examiner with 3 years’ experience in musculoskeletal US imaging on two separate occasions (three images/occasion). Then, the experienced medical doctors with 3 years’ experience in musculoskeletal US imaging conducted the US examinations in CTS.

### 2.4. Ultrasound Image Analysis

The subjects were in a supine position with the elbow extended, forearm supinated and shoulder in a neutral position. The probe was placed in the short-axis view at the carpal tunnel (area between the scaphoid and the pisiform tubercle; these two landmarks were easily identifiable in all patients) [14]. Then, the probe was placed in the short-axis view on the mid-forearm (where the MN leaves the ulnar artery and travels in the fascial plane between the flexor digitorum superficialis and flexor digitorum profundus muscles). Three cross-sectional US images were obtained to measure the EI of PA, the MN at the carpal tunnel, the EI of MS, and the ‘PA and MN’ at the mid-forearm in a neutral position, respectively. Then, cross-sectional images were taken after a whole-hand grasp movement, to evaluate MN transversal displacement. The whole-hand grasp movement means that the wrist was kept in the neutral position while all four fingers and thumb moved from full finger extension to maximum flexion to make a fist [15].

**Figure 1:**
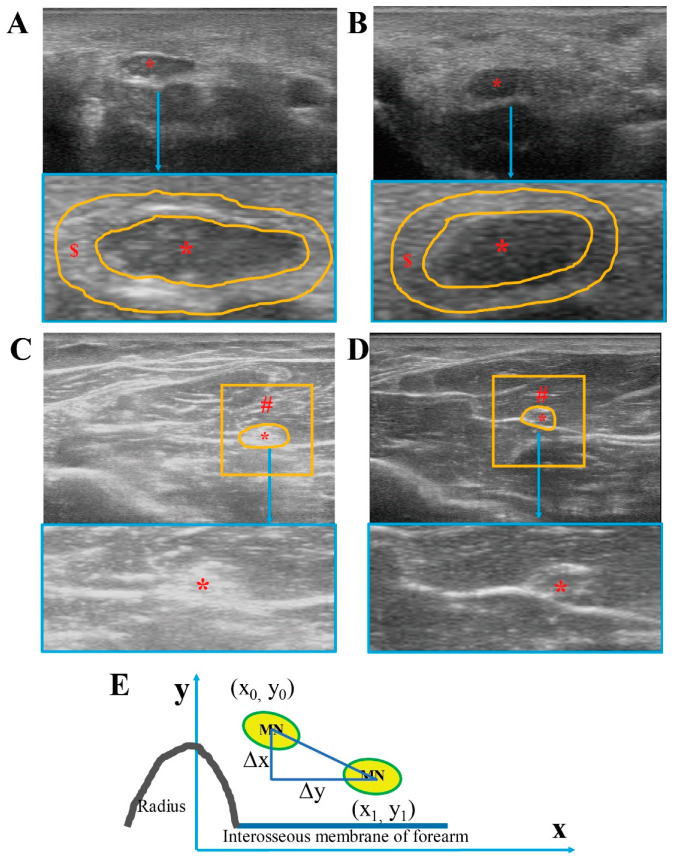
Ultrasound images of median nerve and surrounding tissue. Ultrasound images from CTS subjects at carpal tunnel (**A**) and at mid-forearm (**C**). Ultrasound images from healthy controls at carpal tunnel (**B**) and at mid-forearm (**D**). MN displacement was measured at mid-forearm as the difference between the centroid coordinates $\sqrt{\left({\Delta x}_{-}^{2}{\Delta y}^{2}\right)}$ (**E**). * = median nerve, $ = paraneural area (PA) indicated by the orange line in A, B, # = region of interest myofascial structure around the median nerve (1 cm^2^) except the median nerve and paraneural area.

Three 8-bit grey-level US images per subject at each segment (carpal tunnel and mid-forearm) were analyzed with ImageJ software [16]. In each image, the PA regions, MN at carpal tunnel, MS region, ‘PA and MN’ at mid-forearm, and their mean grey values were selected and measured. The range between 0 (black) and 255 (white) was used to estimate the EI of the selected region. At the carpal tunnel segment, the inner boundary of the PA was defined between the nerve fascicles and the epineurium; the outer boundary of the PA was defined along the boundary of the tendon around the MN. For the MN fascicles, the boundary was defined as between the nerve fascicles and the epineurium. At the mid-forearm segment, 1 cm^2^ regions of interest of MS around the MN (1 cm × 1 cm) were selected and measured, including the flexor digitorum superficialis, flexor digitorum profundus, and the fascial plane between them, except the ‘PA and MN’. The ‘PA and MN’ were estimated together at the mid-forearm, since it is impossible to distinguish between the ‘PA and MN’ on the US image (Figure 1A–D). The data obtained from the three images for each subject were then averaged to obtain the representative value of EI of PA, MN at carpal tunnel and MS, and ‘PA and MN’ at mid-forearm, respectively. 

Regarding the MN transversal displacement, the position of the MN was defined as the centroid coordinates of MN in the images of the neutral position and of after the whole-hand grasp movement. At the carpal tunnel segment, it was calculated that the distance between the centroid coordinates of MN and the imaginary lines of the scaphoid bone were landmarks in the ulnar-radial direction (x), and that between the centroid coordinates of MN and the transverse line connecting the tubercles of scaphoid and pisiform in the palmar-dorsal direction (y) in the first and last centroid positions at carpal tunnel. At the mid-forearm segment, the parallel lines of the interosseous membrane of the forearm were landmarked in the ulnar-radial direction (x), whereas, a vertical line perpendicular to the parallel line of the interosseous membrane is made through the highest point of the radius as in the palmar-dorsal direction (y) at the mid-forearm. We calculated the distance of the centroid coordinates of MN in the first case in a neutral position (x_0_, y_0_), and the last centroid positions after a whole-hand grasp movement (x_1_, y_1_) (Figure 1E). MN displacement was measured as the difference between the centroid coordinates: $\sqrt{\left({\Delta x}_{-}^{2}{\Delta y}^{2}\right)}$ [3,15,17]. 

### 2.5. Histological Study 

After excising the skin and subcutaneous tissue, the MN and surrounding tissue (tendons at the carpal tunnel and myofascial structure at the mid-forearm) were taken carefully from nine non-embalmed cadavers for further histological procedure, as our previous study described [3]. All representative samples, after fixing in 10% neutral buffered formalin, were then submitted for dehydration and further clearing prior to paraffin embedding. Cross-sections were cut into 10 μm thicknesses and stained with hematoxylin and eosin, Azan–Mallory and S100 immunohistochemical staining to study the morphological characteristics of the MN and the surrounding tissue. 

### 2.6. Statistical Analysis

All data management and statistical analyses were performed with IBM SPSS software, version 25.0. The Shapiro–Wilks test was performed to study data distribution, firstly to determine whether variables were normally distributed or not. The gender, age, height, weight or mean body mass index (BMI), and all the EIs are reported as means ± standard deviations (M ± SD), since their distribution was normal. The intra-class correlation coefficient (ICC A,1: two-way random, single measures, absolute agreement) was used to quantify inter-rater and intra-rater reliability for the quantitative EI of PA, MN at carpal tunnel, the EI of MS, and the ‘PA and MN’ at the mid-forearm in the control group. Differences in the age, height, weight or mean body mass index (BMI), and EIs between the CTS and control subjects were analyzed according to the unpaired, two-sample t-test. The MN displacement variable was summarized from median, minimum and maximum, since its distribution was not normal. Differences in MN displacement were examined with Wilcoxon’s rank sum test. The relationships between the EI of PA, MN at the carpal tunnel, the EI of MS, the ‘PA and MN’ and the MN displacement were evaluated with Spearman’s correlation coefficient and corresponding *p*-values. A *p*-value of less than 0.05 was always considered as the limit for statistical significance.

## 3. Results

### 3.1. Participant Characteristics

A total of 16 CTS patients and 16 age- and gender-matched control subjects were included in this study. Their characteristics are shown in Table 1. There were no statistically significant differences in gender, age, height, or weight or mean body mass index (BMI) between the CTS and control group (*p* > 0.05). 

### 3.2. Inter-Rater and Intra-Rater Reliability in Control Subjects

Table 2 shows the results of the inter-rater and intra-rater reliability analyses in the control group. The results demonstrated excellent inter-rater and intra-rater reliability for the EI of PA, MN at the carpal tunnel, for the EI of MS, and the ‘PA and MN’ at the mid-forearm according to the ICC values above 0.75 [18]. This proves the validity and high reproducibility of the US assessment for the measures of this study. 

### 3.3. US Parameters between CTS and Control and Correlations between US Parameters and MN Displacement

**Figure 2:**
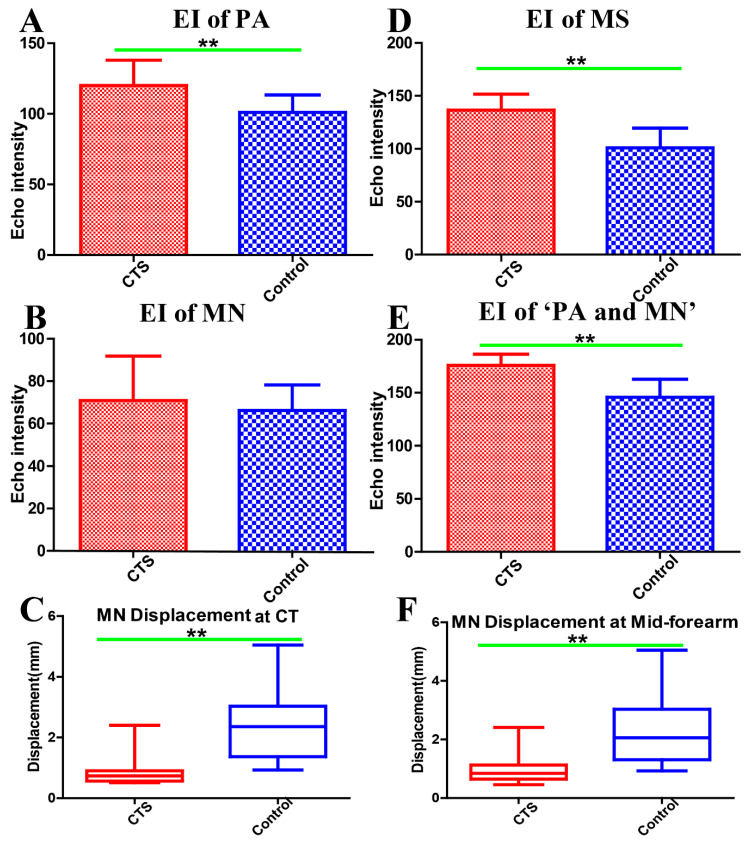
Echo intensity of the paraneural area at the carpal tunnel (**A**). Echo intensity of the median nerve at the carpal tunnel (**B**). Median nerve displacement at the carpal tunnel (**C**). Echo intensity of myofascial structure around the median nerve (1 cm^2^) at the mid-forearm (**D**). Echo intensity of the paraneural area and median nerve at the mid-forearm (**E**). Median nerve displacement at the mid-forearm (**F**). EI: echo intensity, PA: paraneural area, MN: median nerve, CT: carpal tunnel, MS: myofascial structure, CTS: carpal tunnel syndrome, ** Difference between groups is statistically significant (*p* < 0.01).

At the carpal tunnel segment, the EI of the PA was significantly higher in CTS subjects (CTS: 120 ± 17.98; control: 101.1 ± 12.3; *p* = 0.008), whereas there was no significant difference in the EI of the MN (CTS: 70.88 ± 20.96; control: 66.12 ± 12.13; *p* = 0.462) (Figure 2A,B). At the mid-forearm, both the EI of the MS (CTS: 136.56 ± 14.97; control: 100.97 ± 18.55; *p* < 0.001) and the EI of the ‘PA and MN’ (CTS: 175.92 ± 10.45; control: 145.83 ± 16.93; *p* < 0.001) were significantly higher in CTS than in control subjects (Figure 2D,E). MN displacement was significantly reduced in CTS subjects, both at the carpal tunnel and the mid-forearm (Figure 2C,F). In addition, there were negative correlations among the MN transversal displacement with the EI of PA (rs = −0.484, *p* = 0.004) at the carpal tunnel, the EI of MS (rs = −0.479, *p* = 0.002), and the EI of ‘PA and MN’ (rs = −0.605, *p* < 0.001) at the mid-forearm.

## 4. Discussion

In the present study, we used computer-assisted greyscale analysis to measure the EI of PA, the MN at the carpal tunnel, the EI of the MS, and the ‘PA and MN’ at the mid-forearm. The results demonstrated excellent inter-rater and intra-rater reliability for these US parameters in the control group. This is in agreement with previous studies, which have demonstrated that computer-assisted greyscale analysis is a quantitative, reproducible and valid method of analyzing US images of skeletal muscles, and has been used to determine the EI of muscle and MN [11] and to diagnose neuromuscular diseases [10,13]. This proves the validity and high reproducibility of using US to assess and measure these parameters. 

**Figure 3:**
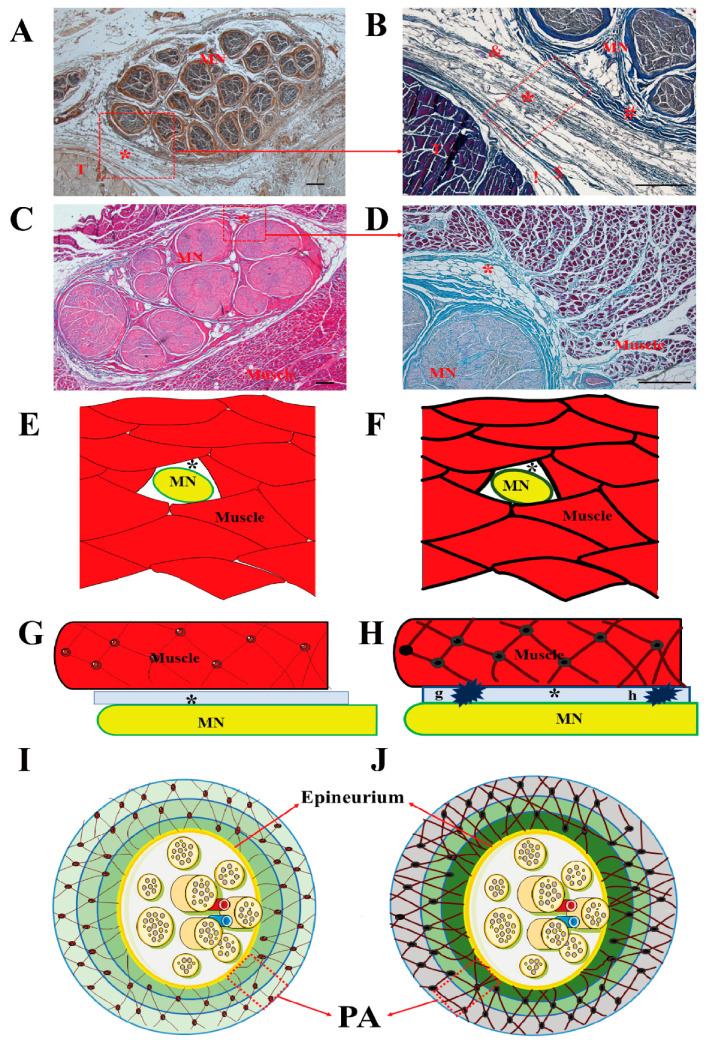
MN and surrounding tissue. Samples stained with S100 antibody (**A**), hematoxylin and eosin (**C**), Azan Mallory Trichrome (**B**,**D**). (**E**,**F**,**G**,**H**): Sketch of median nerve and myofascial structure in cross (**E**,**F**) and longitudinal section (**G**,**H**). Normal myofascial structure in control subjects (**E**,**G**). Disruption of myofascial structure architecture in CTS (**F**,**H**). Compression points of MN (**G**,**H**). Sketch of median nerve and paraneural area (PA) (**I**,**J**). Normal paraneural area in healthy controls (**I**). The disruption of the paraneural area’s architecture in CTS (**J**). MN: median nerve, T: tendon, *: paraneural area, !: epitenon, $: paratenon: &: paraneural sheath, #: epineurium, Scale bar: 100 μm.

The results of our study indicate that the EI of PA at the carpal tunnel, the EI of MS, and the ‘PA and MN’ at the mid-forearm were significantly higher in CTS subjects, whereas there were no significant differences in the EI of the MN itself at the carpal tunnel. The higher EI of ‘PA and MN’ may therefore have been mainly caused by PA, and more so than MN, the at mid-forearm. Pillen et al. found that there was high correlation between interstitial fibrous tissue and EI, which showed that US is a reliable method for ascertaining the severity of structural muscle changes [8], especially for intramuscular and intermuscular connective tissue, including the epimysium, perimysium and endomysium. US is easily transmitted through the normal muscle tissue by echolucent until reflection occurs at the intramuscular and intermuscular connective tissue [19]. However, neuromuscular diseases, such as muscle atrophy, and the replacement of muscle tissue with fat and collagen fibers, make muscle heterogeneous in US imaging [20]. In our previous histological study, we found that the MN cannot be considered by itself, but is embedded in synergic structure with the surrounding myofascial structure that takes part in its movement. The transversal forces act in multiple directions in the myofascial structure, keeping the paraneural sheath equally tensioned, and these are responsible for maintaining the “functional space” in the physiological condition [3]. In fact, the PA is a multilayer structure around MN: there is loose connective tissue in the surrounding extracellular matrix organized in a mesh between the sublayers (Figure 3A–D,I,J). Guimberteau et al. reported the deformation of the micro-vacuolar system during flexor tendon motion [21]. The MN and its connective components are organized in concentric layers which form gliding interfaces between each other, and between the MN and adjacent MS. The deformation of a mesh network within the PA adapts to the intricate movements of the human hand in a similar manner. The gliding interface between the MN and surrounding tissues (intramuscular and intermuscular connective tissue) is an important microenvironment that is essential in minimizing the traction and compression of the MN in response to movement on the transversal axes. In the case of intramuscular or intermuscular connective tissue, and/or muscular, impairment, the synergic structures and coordination may be corrupted [3]. Our results showed that the MN transversal displacement was significantly decreased both at the carpal tunnel and the mid-forearm in CTS subjects, whereas there were negative correlations among the MN transversal displacement with the EI of PA at the carpal tunnel, the EI of MS, and the EI of ‘PA and MN’ at the mid-forearm. The higher EI of MS and PA may suggest that the alteration and disruption of the normal PA and MS architectures, and/or the increased fibrosis around the MN, may reduce the fascial adaptability, and increase the shear stress between the MN and adjacent tissues (Figure 3E–J). For instance, when the upper limb or hand performs a variety of movements, the MN slides according to the various movements to adapt to the changes in the upper limb, and is used to reduce the compression caused by movement or muscle contraction. In this case, changes in paraneural tissue (PA and/or MS) at any point or segment along the path and course of the MN may affect the overall gliding properties of the nerve. In this way, one compression point of the MN may cause reduced mobility along the course of the nerve (Figure 3H), which supports the DCS hypothesis of the coexistence of a distal (carpal tunnel) compression together with a proximal one (mid-forearm). In clinical practice, this may justify the CTS physical therapy and manual therapy not only at the carpal tunnel segment but also along the course of the MN (such as cervical spine, shoulder, elbow, forearm, wrist, and fingers), according to the theory of myofascial continuity of the upper limb [22,23,24].

US assay is therefore a good instrument for CTS diagnosis, especially in the earlier stages, since it can assess PA and MS status and thus indicate a conservative treatment to restore good functioning. However, the gain setting and ‘depth-setting’ must be standardized for each subject, because the EI study is affected by the amount of gain, or depth. In addition, the EI study needs software for good analyses, and a standardization of system settings and parameters to compare EI values with those obtained by varying US devices. Future studies are necessary to clarify the criteria for the EI value of the musculoskeletal system. 

This study had several limitations. First, the numbers of enrolled CTS and control subjects were small. In addition, the incidence of CTS being more than twice as common in females as it is in males [25], it is difficult to generalize these study results to all CTS patients, and further studies with larger sample sizes according to the association of gender with CTS are needed to improve the clinical applicability of this method in neuromuscular diseases. Second, we only investigated the MN displacement on the transversal axes at the carpal tunnel and mid-forearm, and further studies with MN longitudinal displacement need to be performed to better understand the MN gliding on the longitudinal axes.

In conclusion, the EI alteration of PA and MS around the MN in CTS may indicate an increased fibrosis around the MN, which may affect the ability to glide of the nerve, leading to abnormal pressure along the course of the MN. If the abnormal pressure persists, it may cause severe compression and further result in axonal damage. These results may partly explain the roles of PA and MS in CTS pathogenesis. 

## Figures and Tables

**Table 1:** Participant characteristics.

Characteristic	CTS Group	Control Group	*p*-Value
Age (y)	62.87 ± 13.90	59.71 ± 13.33	0.715
Sex	9 F/7 M	9 F/7 M	NA
Height (cm)	168.50 ± 9.91	171.38 ± 6.13	0.474
Weight (kg)	77.00 ± 13.61	78.00 ± 14.01	0.852
BMI (kg/m^2^)	27.19 ± 4.55	26.52 ± 4.63	0.707

Unpaired, two-sample *t*-test. NA: not applicable. Values are presented as numbers or means ± SD. CTS, carpal tunnel syndrome, BMI: body mass index, F: female, M: male.

**Table 2:** Inter-rater and intra-rater reliability of US in control subjects.

		ICC (A,1)	*p*-Value
Inter-rater reliability	EI of PA	0.947	<0.001
	EI of MN	0.892	<0.001
	EI of MS	0.987	<0.001
	EI of ‘PA and MN’	0.920	<0.001
Intra-rater reliability	EI of PA	0.910	<0.001
	EI of MN	0.843	<0.001
	EI of MS	0.989	<0.001
	EI of ‘PA and MN’	0.804	<0.001

EI, echo intensity; ICC (A,1), intraclass correlation coefficient: two-way random, single measures, absolute agreement; MN, median nerve. PA: paraneural area; MS: myofascial structure; PA and MN: paraneural area and median nerve.

## Abbreviations

EI	echo intensity
PA	paraneural area
MN	median nerve
CT	carpal tunnel
MS	myofascial structure
CTS	carpal tunnel syndrome
DCS	double crush syndrome
